# The Experiences and Job Performance of Older Paratransit Drivers

**DOI:** 10.1093/geroni/igaf122.2486

**Published:** 2025-12-31

**Authors:** Shayna Gleason

**Affiliations:** International Transportation Learning Center / Transit Workforce Center, Washington, District of Columbia, United States

## Abstract

Public transportation’s frontline workforce is already considerably older than the U.S. workforce overall, and getting older. Few if any prior studies have examined the performance and experiences of older paratransit drivers, despite their growing prevalence and paratransit’s growing need for qualified drivers of any age. The present study filled this knowledge gap by examining the relationships between driver age and various aspects of job performance, as well as the subjective experiences of older paratransit drivers of their jobs. The research questions for this study were: 1) What is the relationship between driver age and driver job performance? 2) What are older paratransit drivers’ experiences of aging in their positions? Quantitative data from ACCESS Transportation Systems—the Pittsburgh, PA regional paratransit system—were used to answer the first research question. The associations between driver age and frequency of accidents, passenger complaints, and passenger compliments were analyzed using zero-inflated negative binomial regressions. The second research question was answered using qualitative data collected in a semi-structured focus group of eight ACCESS drivers. The quantitative results suggested that age is negatively associated with accidents, compliments, and complaints. The qualitative results indicated a high level of job satisfaction among participants, often driven by altruistic motives, though they acknowledged challenges of the job, some of which they expected to be exacerbated by age-related physical or cognitive changes. The results reveal an important and continuing role for older drivers in paratransit services, and an urgent need to focus on effective recruitment and retention initiatives for this population.

